# Precision of computed tomography and cartilage-reproducing image reconstruction method in generating digital model for potential use in 3D printing of patient-specific radial head prosthesis: a human cadaver study

**DOI:** 10.1186/s41205-021-00093-w

**Published:** 2021-01-28

**Authors:** Suriya Luenam, Theeraset Bantuchai, Arkaphat Kosiyatrakul, Malee Chanpoo, Kantapat Phakdeewisetkul, Chedtha Puncreobutr

**Affiliations:** 1 Department of Orthopaedics, Phramongkutklao Hospital and College of Medicine, 315 Ratchawithi Road, Bangkok, 10400 Thailand; 2 Department of Anatomy, Phramongkutklao Hospital and College of Medicine, Bangkok, Thailand; 3 Biomechanics Research Center, Meticuly Co. Ltd., Chulalongkorn University, Bangkok, Thailand; 4 Advanced Materials Analysis Research Unit, Department of Metallurgical Engineering, Faculty of Engineering, Chulalongkorn University, Bangkok, Thailand

**Keywords:** Radial head, Prosthesis, 3D printing, Patient-specific, Cartilage, Image reconstruction

## Abstract

**Background:**

A prosthetic replacement is a standard treatment for an irreparable radial head fracture; however, the surface mismatch of the commercially available designs is concerned for the long-term cartilage wear. The patient-specific implant created from 3D printing technology could be favorable in replicating the normal anatomy and possibly reduce such sequela. Our study aimed to assess the precision of the computed tomography (CT) and cartilage-reproducing image reconstruction method (CIRM) in generating digital models for potentially use in manufacturing the patient-specific prosthesis from 3D printing.

**Methods:**

Eight intact  elbows (3 right and 5 left) from 7 formalin**-**embalmed cadavers (4 males and 3 females) with mean age of 83 years (range, 79–94 years) were used for this study. Computerized 3D models were generated from CT, and CIRM. The cartilage-reproducing image reconstruction method has compensated the cartilage profile based on the distance between the subchondral surfaces of the radial head and surrounding bones in CT images. The models of actual radial head geometry used as the gold standard was generated from CT arthrography (CTA). All models of each specimen were matched by registering the surface area of radial neck along with the tuberosity. The difference of head diameter, head thickness, and articular disc depth among three models was evaluated and analyzed by Friedman ANOVA and multiple comparison test using Bonferroni method for statistical correction. A *p*-value of less than 0.01 was considered statistically  significant. The difference of overall 3D geometry was measured with the root mean square of adjacent point pairs.

**Results:**

The analysis displayed the difference of diameter, thickness, and disc depth across the models (*p*< 0.01). Pairwise comparisons revealed statistically significant difference of all parameters between CTA models and CT models (*p*< 0.01) whereas no difference was found between CTA models and CIRM models. The mean difference of overall 3D geometry between CTA models and CT models was 0.51±0.24 mm, and between CTA models and CIRM models was 0.24±0.10 mm.

**Conclusions:**

CIRM demonstrated encouraging results in reestablish the normal anatomy and could be potentially used in production process of 3D printed patient-specific radial head prosthesis.

3D printing is an additive manufacturing process that uses a 3D digital model to physically build an object in layers [1]. The use of 3D printing is rising and has become more prevalent in treatment of elbow fractures in the recent years [2–4]. Due to the advantage of fabricating objects with complex freeform geometry, 3D printing can produce patient-specific implants based on their specific anatomy [5].

The radial head plays an important role in stabilizing the joint when the elbow injury associated with ligamentous incompetence [6]. In this setting, replacement with the metal prosthesis is generally recommended for unfixable radial head fracture [7, 8]. The surface mismatches between the radial head implants and the anatomic characteristics of the proximal radius decreased radiocapitellar contact areas and increased radiocapitellar contact forces which is potential for the long-term cartilage wear [9–11]. Previous studies on morphology of radial head indicated that precisely replicates individual anatomy is incapable to achieve by any currently available prosthetic designs and the patient-specific implant may be more favorable [8, 12]. The patient-specific implant of radial head including the head component and stem could be generated with computer-aided design and 3D printing technology (Fig. 1).

**Fig. 1:**
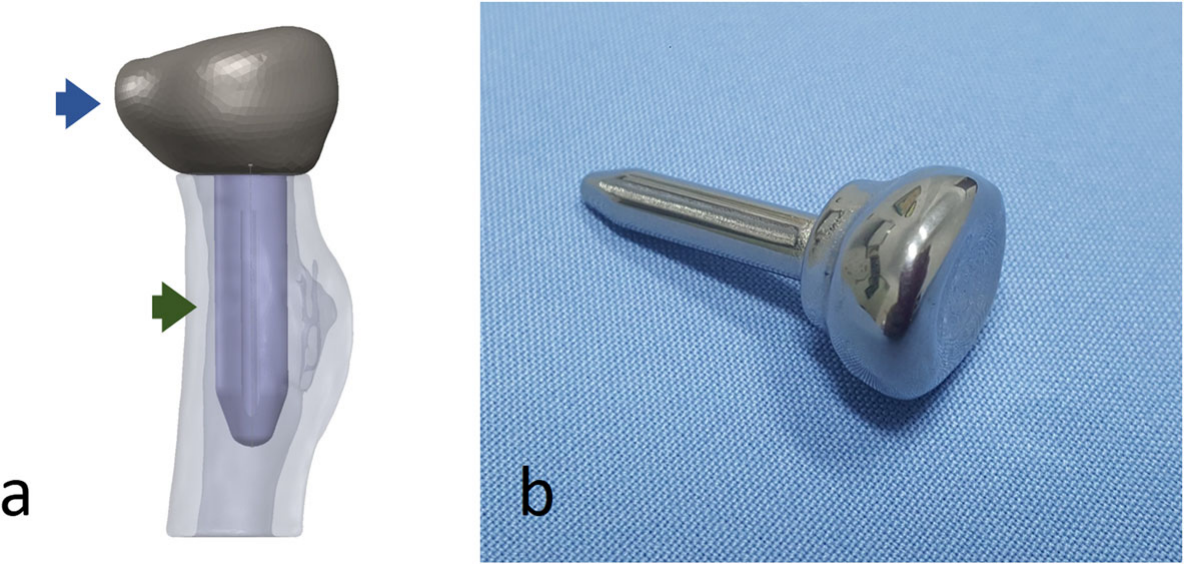
The 3D printed patient-specific radial head prosthesis **a** digital model of a prosthesis consisting of the radial head component (blue arrow) and stem (green arrow) generated with computer-aided design. **b** 3D printed titanium prosthesis manufactured by  selective laser melting process

Computed Tomography (CT) has been routinely used as a standard image reconstruction resource to create 3D digital model for additive manufacturing of custom-made prostheses [12, 13]. The limitation of such method is that the cartilage profile is not incorporated as the CT images mainly show subchondral bone and poorly distinguish the cartilage from the surrounding synovial fluids and soft tissue [14]. Based on the previous studies, the cartilage thickness of the radial head ranged widely from 0 to 3.5 mm and contributed to dish depth and the radius of curvature [15]. For this reason, inclusion of the cartilage outline may be clinically important for the precise design of anatomic implants.

To visualize cartilage dimension, CT arthrography (CTA) which is the combined CT with intraarticular injection of contrast agents or air has been developed [16–18]. Previous studies demonstrated the accurate results of CTA in use for 3D model reconstruction of bone and cartilage surface [18, 19]. However, its clinical use is somewhat limited due to its invasive nature. In this study, we proposed an alternative method to generate the cartilage geometry in the CT images. The cartilage-reproducing image reconstruction method (CIRM) has compensated the cartilage profile based on the distance between the subchondral surfaces of the radial head and surrounding bones.

The objective of this study was to assess the precision of CT and CIRM in generating digital models for potential use in 3D printing of patient-specific prosthesis in comparison to the actual geometry of the radial head which was generated from CTA.

## Material and methods

### Generation of 3D models

**Fig. 2:**
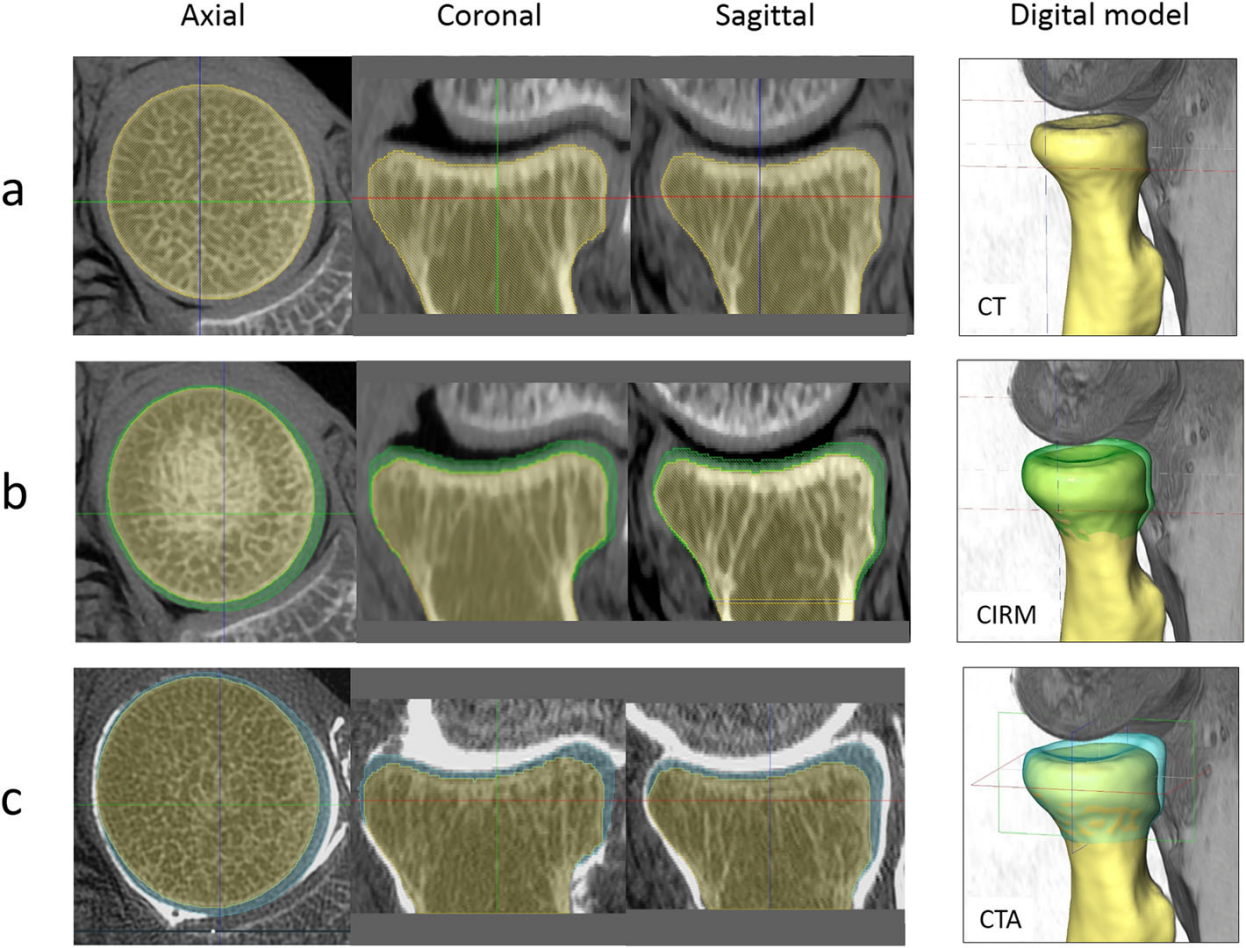
The computerized 3D radial head models of each elbow were generated. **a** 3D model created from segmentation of the CT images (yellow radial head). **b** 3D model created from CT images with cartilage-reproducing image reconstruction method (green radial head). **c** 3D model created  from segmentation of the CT arthrography images (light blue radial head)

The study was approved by and conducted in full compliance with the Royal Thai Army Medical Department Institutional Review Board with number IRBRTA 291/2560. Eight intact elbows (3 right and 5 left) from 7 formalin**-**embalmed cadavers (4 males and 3 females) with mean age of 83 years (range, 79–94 years) were used for this study. Computerized 3D radial head models of each elbow were generated from the CT, CIRM, and CTA in succession (Fig. 2). The details were as follows:

#### Creation of 3D model from CT (3Dmodel-CT)

High-resolution CT scan (Philips Brilliance 64 CT scanner, Cleveland, Ohio, USA; voxel size 0.2 × 0.2 × 0.3 mm3, 120 kV, 150 mAs, pitch 0.5) were performed. The similar resolution values had been previously described for the virtual bone digital model reconstructions in several studies [20–22]. A level of accuracy and reliability of measurement capability that approaches the higher resolution using microCT were reported [23]. The DICOM (Digital Imaging and Communications in Medicine) files obtained from CT scan were used to create the 3D model using Amira-Avizo software (Thermo Fisher Scientific, MA, USA). Segmentation of bone was applied based on Hounsfield unit (range: 250 to 2500). This is the standard value described for segmenting bone in the several studies [14, 19, 24].

#### Creation of 3D model from CIRM (3Dmodel-CIRM)

CIRM was applied to the CT images for compensation of the cartilage profile along the margin of the radial head at the proximal radioulnar and radiocapitellar joints.

**Fig. 3:**
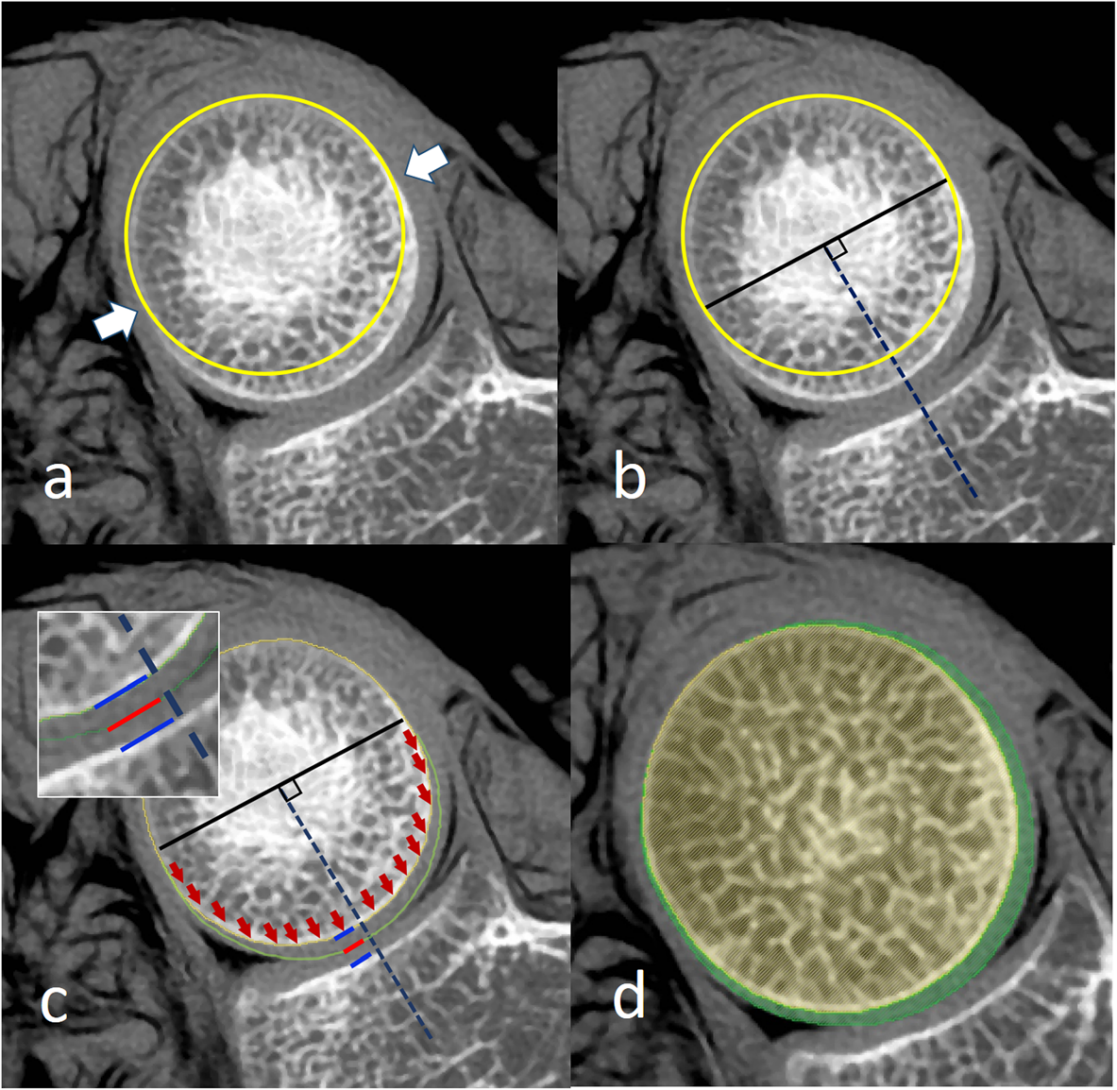
The cartilage-reproducing image reconstruction method of CT image at proximal radioulnar joint. **a** Determine the ANA junctions (white arrow) using an alteration of the circular curve of nonarticular portion. **b** Create a reforming axis (blue dash line) using perpendicular line from the midpoint of the straight line between both ANA junctions. **c** Translate the articular portion along this axis until a reformed border touches half the distance between the subchondral surfaces at the mid portion of radial notch. **d** Fill the area over ANA junctions until the uneven borders are on the same arch. ANA: articular-nonarticular

To compensate for the cartilage geometry articulating with the radial notch of the ulna, an articular portion of the radial head in each axial image was defined. The articular-nonarticular (ANA) junctions were determined using an alteration of the circular curve of nonarticular portion (Fig. 3a). A perpendicular line from the midpoint of the straight line between both ANA junctions was used to establish a reforming axis (Fig. 3b). Volume of the articular portion was magnified by translating the articular portion along this axis until a reformed border touched half the distance between the subchondral surfaces at the mid portion of radial notch (Fig. 3c). The area over ANA junctions was subsequently filled until the uneven borders were on the same arch (Fig. 3d).

**Fig. 4:**
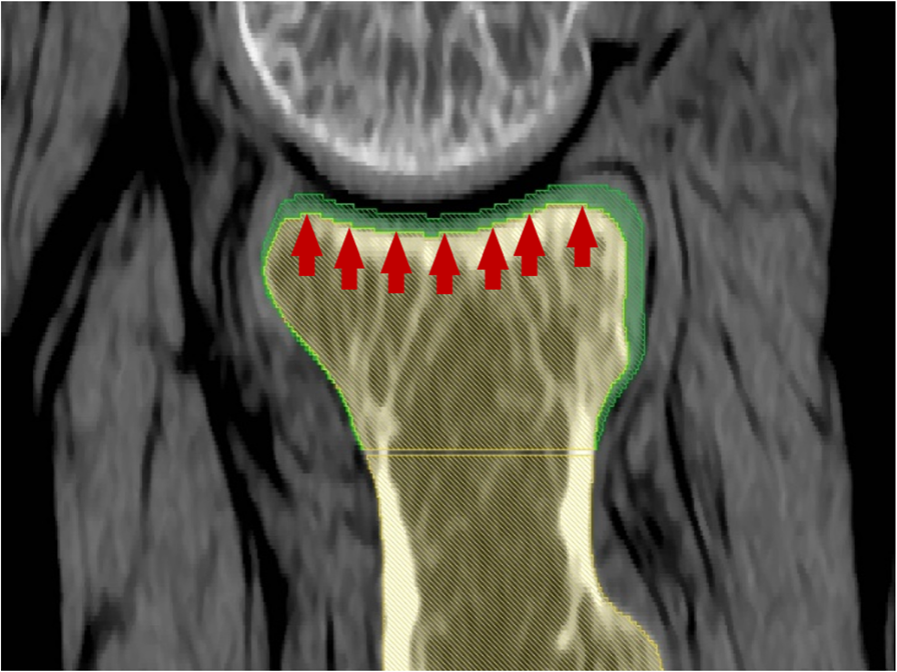
The cartilage-reproducing image reconstruction method of CT image at radiocapitellar joint. Translate the articular portion toward the capitellum until a reformed border  touches half the distance between both subchondral surfaces

To compensate for the cartilage geometry articulating with the capitellum, the articular portion of radial head was translated the articular portion toward the capitellum until a reformed border touched half the distance between both subchondral surfaces (Fig. 4).

#### Creation of 3D model from CTA (3Dmodel-CTA)

A 10 ml solution (5 ml Iopromide (Ultravist 300**,** Schering AG, Berlin, Germany) which is low-osmolar, non-ionic, iodinated contrast medium and 5 ml 0.9% normal saline) was injected into the elbow joint under fluoroscopic guidance. CT scan was subsequently repeated in order to generate the 3D model. The CTA image segmentation method for cartilage described by Allen et al. was used [18].

Reparation and preparation of stereolithography (STL) files of 3D models were done by the software tool MAGICS (Materialise, Leuven, Belgium) for further analysis.

**Fig. 5:**
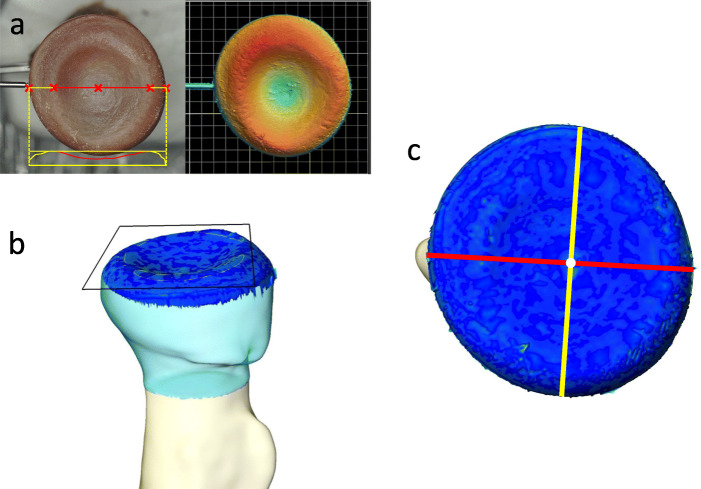
Validation on accuracy of 3Dmodel-CTA*.* **a** Actual surface of radial head was scanned with 3D measuring macroscope. **b** Best-fit plane of the articular rim (black square outline) of 3Dmodel-CTA (light blue) was aligned with the 3D surface profile obtained from surface scanning (dark blue). **c** The center of articular disc (white dot) was defined as the furthest point from the best-fit plane of the articular rim. The coronal diameter (red line) and sagittal diameter (yellow line) were measured across the center to assess the difference between 3Dmodel-CTA and 3D surface profile. 3Dmodel-CTA: 3D model generated from CT arthrography

To evaluate the precision of all digital models which obtained from the same scanning protocol, 3Dmodel-CTA had been set as a gold standard. The accuracy of 3Dmodel-CTA was validated (Fig. 5). Comparison of coronal and sagittal diameters with the actual anatomic geometry obtained from the surface scanning was executed. All radii were dissected and denuded of all soft tissue. Each radial head was scanned on the proximal surface with a 3D measuring microscope (KEYENCE COR VR3200, Itasca, USA) using high magnification mode (Vertical 28 mm and Horizontal 37 mm) with measurement accuracy of ± 2 μm. The microscope utilizes structured light scanning technique to geometrically reconstruct 3D surface. The structured (pattern) light is first emitted from the transmitter lens and projected onto the specimen. When the reflected light is observed from another angle (receiver lens), height differences on the surface make the bands of light appear distorted. An image of these distortions is taken using a CMOS sensor, and triangulation was then used to calculate and measure the radial head topology. To assess the difference of radial head diameters, the best-fit plane of the articular rim and radial tuberosity of the 3D surface profile obtained from the surface scan and 3Dmodel-CTA was aligned. The center of articular disc was defined as the furthest point from the best-fit plane of the articular rim. The coronal diameter was measured along the line across center and the most prominent point of radial tuberosity. The sagittal diameter was measured across the center perpendicular to the coronal plane. No statistically significant difference of the coronal and sagittal diameters was revealed. The mean coronal diameter difference was 0.08±0.19 mm (range, − 0.12 to 0.51 mm) and sagittal diameter difference was 0.06±0.11 mm (range, − 0.29 to 0.06 mm).

### Measurements

#### The diameter, thickness, and articular depth

**Fig. 6:**
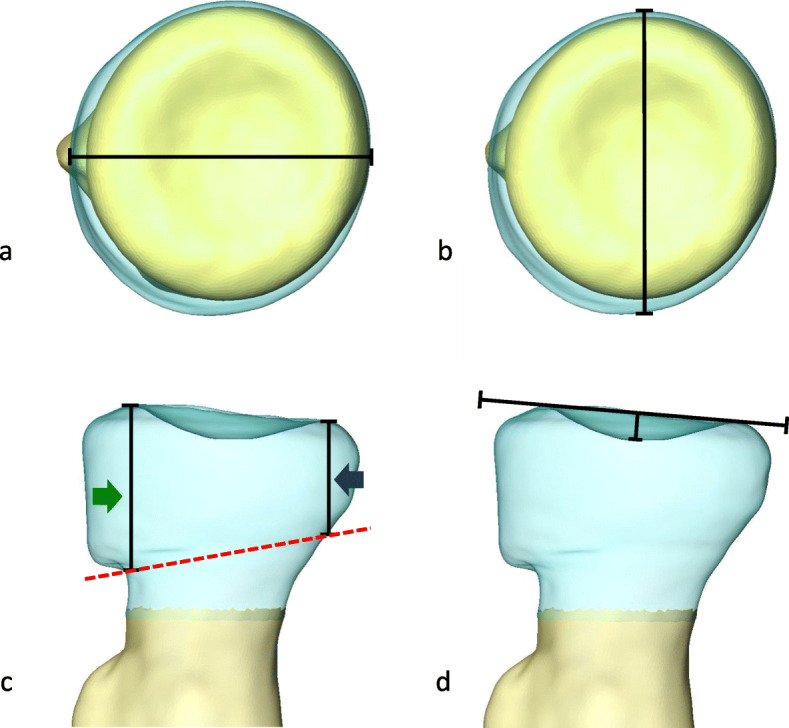
Measurements. **a** Coronal diameter. **b** Sagittal diameter. **c** Thickness at the medial rim (green arrow) and lateral rim (blue arrow) measured from the line across the head neck junction (red dash line) **d** articular disc depth

The coronal, sagittal, and axial planes of each 3Dmodel-CTA were used as references. The coronal plane was formed on the center of the articular disc and the line through the widest bone at the middle portion of radial tuberosity. The center of articular disc was defined as the furthest point from the best-fit plane of the articular rim. The sagittal and axial planes were formed perpendicular to the coronal plane. The surface registration of 3Dmodel-CTA, 3Dmodel-CIRM and 3Dmodel-CT in the same specimen was performed to align all models in matching position for measurements. The area of registration was radial tuberosity and diaphyseal surface. The radial head diameters along coronal and sagittal planes were assessed at the same axial position which had the longest coronal diameter in the 3Dmodel-CTA (Fig. 6a and b). The radial head thickness was assessed in the coronal plane using the distance along sagittal plane from the line across the head neck junction of 3Dmodel-CTA to the most proximal points of the medial and lateral rims (Fig. 6c). The medial and lateral vertexes of head neck junction was determined on the agreement of 2 observers (1 orthopedic surgeon and 1 engineer) using the method described by Puchwein et al. [25]. The vertex was defined by the point of the curve that is farthest from a line between the beginning and end of the curve. The articular disc depth was defined as the shortest distance from furthest point of the articular surface to the line between the most proximal points of medial and lateral rims in the coronal plane (Fig. 6d). All parameters in each specimen were measured automatically using Matlab software (The MathWorks, Natick, MA, USA). The difference of parameters between the models (3Dmodel-CTA and 3Dmodel-CT, 3Dmodel-CTA and 3Dmodel-CIRM, 3Dmodel-CIRM and 3Dmodel-CT) was calculated.

#### The difference of overall 3D geometry

Following the tuberosity-diaphyseal surface registration, the root mean square (RMS) of adjacent point pairs in the area of radial head between the digital models was measured automatically using Amira-Avizo software to demonstrate the difference of overall 3D geometry.

All parameters except overall 3D geometry were measured by 2 observers (1 orthopedic surgeon and 1 engineer) separately. The mean of the two measurements was used for analysis. The interrater reliability was verified using the intraclass correlation coefficient, equal to 0.99 (*p*< 0.001).

### Analysis

For a comparative purpose, the diameter, thickness, and articular depth among the different generating techniques in the same specimen were analyzed by Friedman ANOVA and multiple comparison test. The null hypothesis is the distribution of 3Dmodel-CTA, 3Dmodel-CT and 3Dmodel-CIRM are the same. The pairwise comparisons were performed if the null hypothesis was rejected with the *p*-value less than 0.01. Significant values had been adjusted by Bonferroni correction for multiple tests. A *p*-value of less than 0.01 was considered statistically  significant.

## Results

### The diameter, thickness, and articular depth

The mean, standard deviation, range, and statistical analysis of diameter, thickness, and articular depth are presented in Table 1. Friedman test rejects the null hypothesis in the parameters of coronal diameter (*p*=0.002), sagittal diameter (*p*=0.001), medial thickness (*p*=0.002), lateral thickness (*p*=0.002), and articular depth (*p*=0.002).

**Table 1:** Mean, SD, range and statistical analysis of diameter, thickness, and articular depth

	3Dmodel-CTA	3Dmodel-CT	3Dmodel-CIRM	Friedman test (*p*-value)	Pairwise comparisons (Bonferroni correction)		
	mean ± SD range (mm)	mean ± SD range (mm)	mean ± SD range (mm)		3Dmodel-CTA vs. 3Dmodel-CT (*p*-value)	3Dmodel-CTA vs. 3Dmodel-CIRM (*p*-value)	3Dmodel-CIRM vs. 3Dmodel-CT (*p*-value)
Diameter along coronal plane	21.85±0.99 20.68**–**23.55	20.86±0.94 19.94**–**22.81	21.66±0.91 20.64**–**23.39	0.002*	0.003*	1.000	0.018
Diameter along sagittal plane	22.39±1.52 20.45**–**24.13	21.51±1.43 19.34**–**23.62	22.07±1.49 19.83**–**24.03	0.001*	0.001*	0.401	0.073
Thickness at medial rim	10.98±1.23 9.24**–**12.32	9.91±0.96 8.55**–**11.57	10.75±1.05 9.26–12.30	0.002*	0.003*	1.000	0.018
Thickness at lateral rim	8.46±0.9 6.74**–**9.61	7.5±0.97 5.91**–**9.18	8.35±0.94 6.61**–**9.82	0.002*	0.008*	1.000	0.008*
Articular disc depth	2.03±0.20 1.71**–**2.38	1.63±0.19 1.35–1.91	1.75±0.12 1.58**–**1.94	0.002*	0.001*	0.037	0.952

*significant at *p*-value < 0.01

*3Dmodel-CTA* 3D model from CT arthrography, *3Dmodel-CT* 3D model from CT scan, *3Dmodel-CIRM* 3D model from cartilage-reproducing image reconstruction method

The pairwise comparisons (Bonferroni correction) displayed the statistically significant difference of coronal diameter (*p*=0.003), sagittal diameter (*p*=0.001), medial thickness (*p*=0.0023), lateral thickness (*p*=0.008), and articular depth (*p*=0.001) between 3Dmodel-CTA and 3Dmodel-CT. The difference of coronal diameter (*p*=1.000), sagittal diameter (*p*=0.401), medial thickness (*p*=1.000), lateral thickness (*p*=1.000), and articular depth (*p*=0.037) between 3Dmodel-CTA and 3Dmodel-CIRM was not statistically significant.

The difference of all parameters between each pair of generating techniques are presented in Table 2. Between 3Dmodel-CTA and 3Dmodel-CT, the mean coronal diameter difference was 0.99±0.45 mm (range, 0.55 to 1.55 mm) and sagittal diameter difference was 0.88±0.55 mm (range, 0.14 to 1.69 mm). The mean medial thickness difference was 1.07±0.60 mm (range, 0.50 to 2.18 mm) and lateral thickness difference was 0.96±0.61 mm (range, 0.19 to 2.08 mm). The mean articular depth difference was 0.40±0.20 mm (range, 0.11 to 0.63 mm).

**Table 2:** The difference of all parameters between each pair of generating techniques

	Diameter along coronal plane (mm)	Diameter along sagittal plane (mm)	Thickness at medial rim (mm)	Thickness at lateral rim (mm)	Articular disc depth (mm)	Overall geometry (mm)
Difference	mean ± SD (range)	mean ± SD (range)	mean ± SD (range)	mean ± SD (range)	mean ± SD (range)	mean ± SD (range)
3Dmodel-CTA vs. 3Dmodel-CT	0.99±0.4 (0.55–1.55)	0.88±0.55 (0.14–1.69)	1.07±0.60 (0.50–2.18)	0.96±0.61 (0.19–2.08)	0.40±0.20 (0.11–0.63)	0.51±0.24 (0.22–0.89)
3Dmodel-CTA vs. 3Dmodel-CIRM	0.19±0.33 (− 0.20–0.74)	0.33±0.55 (− 0.07–0.90)	0.24±0.39 (− 0.11–1.01)	0.12±0.39 (− 0.27–0.8)	0.28±0.20 (0.08–0.69)	0.24±0.10 (0.14–0.40)
3Dmodel-CIRM vs. 3Dmodel-CT	0.8±0.21 (0.51–1.08)	0.55±0.24 (0.21–1.01)	0.83±0.27 (0.58–1.20)	0.84±0.27 (0.37–1.28)	0.12±0.18 (− 0.12–0.41)	0.44±0.12 (0.27–0.60)

*3Dmodel-CTA* 3D model from CT arthrography, *3Dmodel-CT* 3D model from CT scan, *3Dmodel-CIRM* 3D model from cartilage-reproducing image reconstruction method

Between 3Dmodel-CTA and 3Dmodel-CIRM, the mean coronal diameter difference was 0.19±0.33 mm (range, − 0.20 to 0.74 mm) and sagittal diameter difference was 0.33±0.55 mm (range, − 0.07 to 0.90 mm). The mean medial thickness difference was 0.12±0.39 mm (range, − 0.11 to 1.01 mm) and lateral thickness difference was 0.12±0.39 mm (range, − 0.27 to 0.8 mm). The mean articular depth difference was 0.28±0.20 mm (range, 0.08 to 0.69 mm).

### The difference of overall 3D geometry

**Fig. 7:**
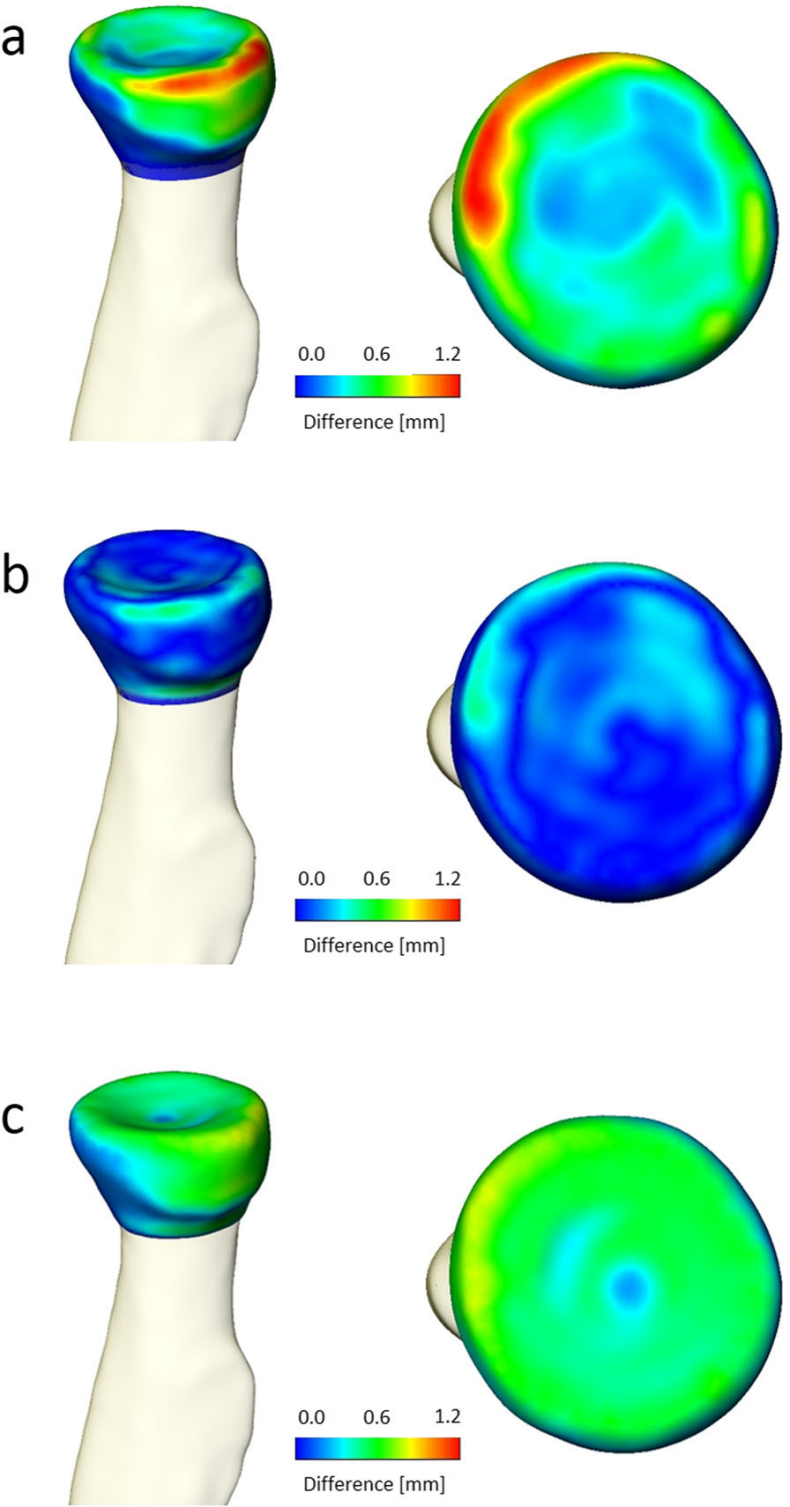
The difference of overall 3D geometry between the models measured with the root mean square (RMS) of adjacent point pairs **a** comparison of 3Dmodel-CTA and 3Dmodel-CT **b** comparison of 3Dmodel-CTA and 3Dmodel-CIRM **c** comparison of 3Dmodel-CIRM and 3Dmodel-CT. The difference is shown in colormap

The difference of overall 3D geometry is presented in Fig. 7. The mean difference of overall 3D geometry between 3Dmodel-CTA and 3Dmodel-CT was 0.51±0.24 mm (range, 0.22 to 0.89 mm). The mean difference between 3Dmodel-CTA and 3Dmodel-CIRM was 0.24±0.10 mm (range, 0.14 to 0.40 mm).

## Discussion

With the advance of metal 3D printing, the digital model can be potentially used as a resource for manufacturing the patient-specific radial head prosthesis [5]. In an attempt to develop a precise head component, this investigation focused on the effectiveness of different image reconstruction methods in creating digital models which is an essential part of the manufacturing process. The cartilage-reproducing image reconstruction method has provided the encouraging results in restoration the true geometric parameters of radial head.

The recent study verified that the surface geometry of proximal radius can be accurately reconstructed based on the obtained geometric features of the contra lateral side [20]. Based on this finding, implantation of the prosthesis in an anatomic position can be planned following the surface registration of the radial tuberosity and diaphyseal area. The stem in conforming to the canal and the neck cut in corresponding to the head component can be planned by means of the computer-aided design and fabricated with 3D printing [13]. Via this method, the location and orientation of the neck cut can be determined or changed without restriction because effectiveness in restoration of the original anatomy is mainly relied on the precision of head component in replicating the articular geometry proximal to the location neck cut. In the present study, the experiment had been set up corresponding to this concept. The key parameters which were typically described in the previous studies were measured. The different (coronal and sagittal) diameter is correlated to the alteration of proximal radioulnar articular geometry. The different (medial and lateral) thickness and articular depth are related to the alteration of radiocapitellar articular geometry. The fixed references obtained from 3Dmodel-CTA were used to assess the parameters of the other models after tuberosity-diaphyseal registration. Although locating of the fixed references among specimens might be inconsistent from the indistinguishable bony structures, comparison of the image reconstruction methods which was executed in the same specimen should represent their efficacy.

In the present study, statistically significant difference of all parameters between the “gold standard” CTA models which is representing the actual geometry and CT models was revealed. The discrepancy of diameters in the axial plane ranging from 0.14 to 1.69 mm could be explained by the inability to visualize circumferential cartilage thickness in CT images. This finding corresponds with the previous anatomic studies which indicated that the cartilage covers the articular surface of the radial head affects its size and morphology [14, 15]. Gianicular et al. described the circumferential cartilage thickness in MRI study ranges widely from 0 to 3.5 mm [15]. Our study displayed the discrepancy in coronal and sagittal planes which may relate to the presence of rim cartilage thickness ranging from 0.19 to 2.18. Yeung et al. reported the narrower range from 0.76 to 1.73 mm [14]. These authors also found that the cartilage thickness across the radial head was unevenly distributed and different between individuals. A statistically significant difference with lower value of the disc depth of 3Dmodel-CT in all specimens was observed in our study. This finding confirmed the increased cartilage thickness around the rim circumference affects the depth of the articular dish [14, 26, 27]. The statistically significant difference of the mentioned parameters suggested that the precision in created the 3D model by means of CT investigations is inefficient.

To improve the visualization of indistinguishable cartilage dimension in CT scan, the alternative assessment with CTA was described [16–18]. Several studies demonstrated the promising accuracy of 3Dmodel-CTA in cartilage thickness measurement [17–19]. However, its clinical use is somewhat limited due to its invasive nature [16]. To mitigate the procedure of intraarticular contrast injection, an alternative of CIRM that uses purely the CT data was proposed. The difference between the models created with this technique and 3Dmodel-CTA was not statistically significant. A mean error of diameters less than 0.34 mm, thickness less than 0.25 mm, disc depth less than 0.29 mm, and overall 3D geometry less than 0.25 mm compared with the gold standard was revealed. Based on the results in this experiment, CIRM could conceivably be used as a viable option in creating a patient-specific radial head prosthesis with the optimal geometry.

There are some limitations in our study. First, the sample size is the relatively small. This may compromise the extent of the variability. However, the sample size calculation based on  the current literature which has the similar assessment of morphologic parameters suggests that the sample is sufficiently representative [28]. Second, the errors from image acquisition and processing for creating CT-based 3D models may occur. The accuracy and precision of a final digital 3D model related to several factors and parameters of the data acquisition process and the subsequent data conversion steps. Noser et al. assessed the accuracy of a processing pipeline for creating CT-based virtual bone models [29]. Typical errors have about the same size as the scan resolution. On the other hand, it has been proved in several studies that linear, angular, and volumetric measurements of 3D models created from the high resolution CT scans are reliable and accurately correspond to the original bones [30–32]. Third, 3Dmodel-CTA had been used as a gold standard to represent the actual anatomy instead of the model created directly from specimen. To verify its accuracy was sufficient, 3Dmodel-CTA was validated by which compared to actual geometry obtained from the surface scanning. A previous in-vitro comparison also evidenced the high accuracy with minimal discrepancy between CTA models and the actual radial head anatomy [28]. Forth, the formalin-embalmed cadavers had been used in the present study. Although formalin fixation may contribute to the alteration of cartilaginous biologic properties, several studies have reported that it has non-measurable effects on cartilage thickness or on the intraarticular geometric configuration [19, 22, 33].

## Conclusion

The present study demonstrated that CIRM demonstrated favorable results in restoration of the diameter, thickness, articular depth and overall 3D geometry in radial head models. Such method could improve the capability to replicate the normal anatomy in generating digital model for potential use in 3D printing of the patient-specific radial head prosthesis. However, the biomechanical tests regarding the elbow kinematic and joint contact pressure following replacement of the implant developed from this method remains largely unknown and should be further studied.

## Data Availability

All data generated during the current study are available from the corresponding author on reasonable request.

## Abbreviations

*3D*: Three-dimensional
*CT*: Computed tomography
*CTA*: CT arthrography
*CIRM*: Cartilage-reproducing image reconstruction method
*3Dmodel-CT*: 3D model generated from CT
*3Dmodel-CIRM*: 3D model generated from CIRM
*3Dmodel-CTA*: 3D model generated from CTA
*STL*: Stereolithography
*DICOM*: Digital imaging and communications in medicine
